# Prevention in Practice – introduction to the supplement

**DOI:** 10.1186/1472-6831-15-S1-S1

**Published:** 2015-09-15

**Authors:** Angelo Mariotti, Iain A Pretty

**Affiliations:** 1 Division of Periodontology, College of Dentistry, The Ohio State University, Columbus, Ohio, 43210, USA; 2 Dental Health Unit, School of Dentistry, University of Manchester, Manchester, England, UK

## 

It is with great pleasure that we present, on behalf of the authors, the manuscripts from the Prevention in Practice Conference held in Cape Town, South Africa, in June 2014. The purpose of the conference was to bring together key opinion leaders in prevention, service design and organization, public health and health economics to discuss the possibilities of, and barriers to, the implementation of effective prevention in general dental practices.

The conference followed a structure that first presented the possible, i.e. those disease entities that can be prevented in practice, how those diseases can be detected and the evidence-based therapies that could be leveraged to achieve health outcomes. We followed these presentations examining how this might be achieved considering work force, economics and practical examples.

Over 100 attendees were present at the conference, representing a wide range of interests and discussions were lively and informative. At the conclusion of the conference it was agreed by the presenters that the collation of the material, including those comments and suggestions from the attendees, should be made accessible to a wider audience. With the kind support of Colgate Palmolive, this special edition of BMC Oral Health has been produced, enabling widespread, free access to the proceedings of this conference.

We hope you find the contents useful, instructive and relevant to your field of practice.

## Competing interest statement

Both authors were funded by Colgate Palmolive to attend the Cape Town Conference. Pretty receives an unrestricted research grant from Colgate Palmolive. Colgate Palmolive did not review, edit or preview the content from this or any other submission in relation to the Prevention in Practice supplement.

## Declarations

This article has been published as part of *BMC Health Services Research* Volume 15 Supplement 1, 2015: Proceedings of Prevention in practice - making it happen. The full contents of the supplement are available online at http://www.biomedcentral.com/bmchealthservres/supplements/15/S1. Publication charges for this supplement were funded by Colgate Palmolive.

